# Promoter Hypermethylation Analysis of Host Genes in Cervical Cancer Patients With and Without Human Immunodeficiency Virus in Botswana

**DOI:** 10.3389/fonc.2021.560296

**Published:** 2021-02-26

**Authors:** Leabaneng Tawe, Surbhi Grover, Nicola Zetola, Erle S. Robertson, Simani Gaseitsiwe, Sikhulile Moyo, Ishmael Kasvosve, Giacomo M. Paganotti, Mohan Narasimhamurthy

**Affiliations:** ^1^Department of Medical Laboratory Sciences, Faculty of Health Sciences, University of Botswana, Gaborone, Botswana; ^2^Botswana-University of Pennsylvania Partnership, Gaborone, Botswana; ^3^Department of Radiation Oncology, Perelman School of Medicine, University of Pennsylvania, Philadelphia, PA, United States; ^4^Department of Otorhinolaryngology-Head and Neck Surgery, and the Tumor Virology Program, Abramson Comprehensive Cancer Center, Perelman School of Medicine, University of Pennsylvania, Philadelphia, PA, United States; ^5^Botswana Harvard AIDS Institute Partnership, Gaborone, Botswana; ^6^Department of Immunology and Infectious Diseases, Harvard School of Public Health, Boston, MA, United States; ^7^Division of Infectious Diseases, Perelman School of Medicine, University of Pennsylvania, Philadelphia, PA, United States; ^8^Department of Biomedical Sciences, Faculty of Medicine, University of Botswana, Gaborone, Botswana; ^9^Department of Pathology, Faculty of Medicine, University of Botswana, Gaborone, Botswana

**Keywords:** Botswana, invasive cervical cancer, human immunodeficiency virus, DNA methylation, tumor supressor gene, human papillomavirus

## Abstract

**Background:** Epidemics of human immunodeficiency virus (HIV) and cervical cancer are interconnected. DNA hypermethylation of host genes' promoter in cervical lesions has also been recognized as a contributor to cervical cancer progression.

**Methods:** For this purpose we analyzed promoter methylation of four tumor suppressor genes (*RARB, CADM1, DAPK1* and *PAX1*) and explored their possible association with cervical cancer in Botswana among women of known HIV status. Overall, 228 cervical specimens (128 cervical cancers and 100 non-cancer subjects) were used. Yates-corrected chi-square test and Fisher's exact test were used to explore the association of promoter methylation for each host gene and cancer status. Subsequently, a logistic regression analysis was performed to find which factors, HIV status, high risk-HPV genotypes, patient's age and promoter methylation, were associated with the following dependent variables: cancer status, cervical cancer stage and promoter methylation rate.

**Results:** In patients with cervical cancer the rate of promoter methylation observed was greater than 64% in all the genes studied. Analysis also showed a higher risk of cervical cancer according to the increased number of methylated promoter genes (OR = 6.20; 95% CI: 3.66–10.51; *P* < 0.001). *RARB* methylation showed the strongest association with cervical cancer compared to other genes (OR = 15.25; 95% CI: 6.06–40.0; *P* < 0.001). Cervical cancer and promoter methylation of *RARB* and *DAPK1* genes were associated with increasing age (OR = 1.12; 95% CI: 1.01-1.26; *P* = 0.037 and OR = 1.05; 95% CI: 1.00-1.10; *P* = 0.040). The presence of epigenetic changes at those genes appeared to be independent of HIV status among subjects with cervical cancer. Moreover, we found that cervical cancer stage was influenced by *RARB* (χ^2^= 7.32; *P* = 0.002) and *CADM1* (χ^2^=12.68; *P* = 0.013) hypermethylation, and HIV status (χ^2^= 19.93; *P* = 0.001).

**Conclusion:** This study confirms the association between invasive cervical cancer and promoter gene methylation of tumor suppressing genes at the site of cancer. HIV infection did not show any association to methylation changes in this group of cervical cancer patients from Botswana. Further studies are needed to better understand the role of HIV in methylation of host genes among cancer subjects leading to cervical cancer progression.

## Introduction

Cervical cancer remains one of the most common cancers affecting females in low and middle-income countries, where 85% of the estimated 570,000 global annual cases occur (1). The burden of disease is greatest in Africa due to a high prevalence of human immunodeficiency virus (HIV) and is increasing rapidly despite wide usage of antiretroviral therapy (ART) (2, 3). Cervical cancer mortality rates are high in women in sub-Saharan Africa including Botswana (1, 4, 5). Human papillomavirus (HPV) infection has been shown to play a crucial role in the development of cervical cancer (6). Human papillomavirus is one of the most common sexually transmitted pathogens, with ~80% of women becoming infected at some point in their lives (7). In ~20% of the infections, the virus is able to persist in epithelial cells and induce pathological changes in the cervix, ranging from dysplasia to high-grade cervical intraepithelial neoplasia (CIN). Women infected with HIV still have relatively high rates of HPV infection and persistence, with subsequent risk of cell transformation and progression to cervical cancer (8). A number of studies performed in Botswana suggest that HIV might influence the distribution of some HPV genotypes (9–13). In one study, a significant association in the prevalence of HPV-16 genotype among HIV-infected patients was reported, despite over 90% of the patients taking ART treatment at the time of cervical cancer diagnosis (13). However, it is generally accepted that persistent high-risk HPV genotypes are necessary, but not always sufficient, to develop cervical cancer (6). Since only a small fraction of HPV-infected CIN lesions progresses to invasive cervical cancer, several studies have indicated that in addition to HPV, host factors, specifically epigenetic changes, play a role in cervical carcinogenesis (14–16). It is known that alterations in DNA methylation are associated with the host genomic response to HIV infection (17, 18) including premature aging and disease progression (19). Furthermore, HIV is known to modify the expression of regulatory and cell-cycle proteins in the cervix of HIV/HPV co-infected women (20–22). It has also been discovered that both post-translational modifications of histones and DNA methylation at specific loci may be involved in cervical cancer development, leading to uncontrolled cell proliferation (23).

In general, epigenetic regulation of gene expression is a vital process that determines the profile of proteins required to ensure the proper and timely occurrence of cellular processes including development, differentiation, organogenesis, stress response, and programmed cell death (24). One of the most widely studied epigenetic mechanisms is DNA methylation, a reversible reaction catalyzed by DNA methyltransferases (DNMTs) (25, 26). Increased DNA methylation in CpG islands (DNA regions that contain a large quantity of CG repeats) has been shown to be associated with increasing persistence of high-risk HPV genotypes (27), severity of CIN lesions (28) and risk of invasive cancer (29). Among the genes that are candidates as markers of cervical cancer risk there are genes involved in cell-cycle control and tissue differentiation regulation (Retinoic Acid Receptor Beta, *RARB*); that are positive mediator of programmed cell death (apoptosis) (Death Associated Protein Kinase 1, *DAPK1*); that encode a member of the immunoglobulin superfamily and is one of the crucial tumor suppressors involved in cell adhesion (Cell Adhesion Molecule 1, *CADM1*); and a transcriptional activator involved in developmental processes (Paired Box 1, *PAX1*). To date, there are a limited number of pertinent primary publications assessing DNA methylation status in patients with cervical cancer from Africa. Furthermore, no studies have been explicitly done to evaluate the aberrantly methylated tumor suppressor genes in the high HIV prevalence setting of Botswana. Therefore, our study aimed to characterize methylation status of four host genes (*RARB, CADM1, DAPK1*, and *PAX1*) that have been studied to a great extent in relation to cervical cancer (28, 30–35). We then explored their possible association with invasive cervical cancer in HIV-infected and uninfected women in Botswana with and without invasive cervical cancer. This may provide insights into potential therapeutic avenues, especially since DNA methylation could potentially be targeted using methylation inhibitors (36, 37).

## Materials and Methods

### Study Design

The study sample comprised of specimens obtained from subjects with (*n* = 128) and without (*n* = 100) cancer. We used the tumor and normal tissue samples available at the National Health Laboratory (NHL) in Gaborone. All the tissue samples archived has been stored at room temperature. The extracted DNA was stored at −20°C prior to analysis. All samples were selected and confirmed according to histological analysis. The demographic and clinical characteristics of patients with invasive cervical cancer have been previously described (13). However, for control specimens, age was not accessible and data on HIV status was incomplete. No outliers were excluded from the analysis. On the basis of developed methods, each experiment was conducted at least twice, with similar results being archived each time.

### Sample Selection and Clinical Characteristics

For this analysis, we used tumor and normal tissue from archived patients' materials. The retrospective, cross-sectional study used formalin-fixed paraffin-embedded (FFPE) tissues from patients with histological confirmation of invasive cervical cancer (cases) and from subjects who underwent routine screening and diagnosed non-cancer (controls). Exclusion criteria among control subjects included: a history of cervical neoplasia, skin or genital warts, the presence of other cancers and past surgery of the uterine cervix. All the study patients were Batswana women, and all invasive cervical cancer diagnoses were made by a pathologist at the NHL in Gaborone, whenever possible, patient demographics, clinical data and HIV status were extracted from medical records through accessing the Intergraded Patients Management System.

### DNA Extraction, High-Risk HPV Detection, and Methylation-Specific PCR Analysis

DNA was extracted from FFPE cervical specimens using an established protocol (38). DNA concentration was measured and quality assessed. The presence of high-risk HPV DNA (HPV-16, HPV-18, and *other* high-risk genotypes, alone or in combination) in the tissue specimen of women with invasive cervical cancer was detected using Abbot real-time PCR (Abbot molecular Inc., Chicago). Extracted host genomic DNA was first subjected to bisulfite treatment using the EZ DNA Methylation Kit (Zymo Research, Irvine) following the manufacturer's instruction. Bisulfite treated DNA was used to analyze the promoter methylation regions of four tumor suppressor genes (*RARB, CADM1, DAPK1*, and *PAX1*) by methylation-specific PCR (MSP), which is the gold standard method of DNA methylation evaluation (34). Supplementary Figure 1 shows a graphic representation of the regions used for bisulfite based methylation measurement for the aforementioned host genes. Two sets of primers (for methylated and unmethylated DNA, respectively) were adopted and applied for each of the four genes examined. See Supplementary Tables 1, 2 for primer sequences and related MSP conditions. Methylation-specific PCR reactions were adopted as previously described (39–42) and modified into a touch-down PCR approach. PCR was then performed using the aforementioned touch-down parameters (see Supplementary Tables 2A,B). Touch-down PCR was designed and used due to its ability to amplify degraded DNA associated with formalin fixation and long-term storage in paraffin. PCR products were run on an agarose gel, and the results are reported as methylated, unmethylated, or a mixture of both at the target DNA sequence (34–43). Methylation-specific PCR was performed twice on all specimens with a third repeat performed if discrepant results were obtained from the first two runs. Two laboratory technicians not associated with the study who were blinded to the histological diagnosis independently read the MSP results.

### Statistical Analysis

Three classes of methylation status: fully unmethylated (U), fully methylated (M) and semi-methylated promoter (MU), were used for all the analyses. When necessary [M + MU] were combined together. We first assessed rates of promoter methylation for each gene and cancer status (presence vs. absence) using Yates-corrected chi-square test and Fisher's exact test (when at least one of the frequency classes was <5). Subsequently, we ran a logistic regression analysis to test which factors were associated to the following dependent variables: cancer status, cancer stage (I–IV), and promoter methylation rate. Factors included: HIV status, high-risk HPV genotypes, patient's age (when available), and promoter methylation rate, when the dependent variable was either cancer status or cancer stage. Data analysis was carried out using Statistical Package for Social Sciences (SPSS) version 20 (IBM). Odds ratios (ORs) and 95% confidence intervals (CI) were calculated. Finally, we evaluated, through Binary Logistic Regression analysis, the possible influence of the number of methylated promoter sites on cancer status. We stratified the methylation data into 5 classes, according to the number of methylated promoter signals (0, 1, 2, 3, and 4).

## Results

### Demographic and Clinical Characteristics of Study Patients

Characteristics of study participants are summarized in Table 1. All available tissue samples from women with a histologically confirmed diagnosis of invasive cervical cancer from the previous study (13) were included (*n* = 128), while the control group (non-cancer subjects) was added (*n* = 100; see Table 1). All samples studied, excluding 7 (from non-cancer control group), were positive for high-risk HPV genotypes. Of 128 specimens from invasive cervical cancer patients, 77 (62.6%) were from HIV-infected women and 46 (37.4%) were from HIV-uninfected women. The HIV-infected patients were younger than their HIV-uninfected counterparts (average age of 43 vs. 61 years, respectively; *P* < 0.001) in patients with invasive cervical cancer (13). Among the non-cancer control group, 24 (24.0%) were HIV-uninfected, while 10 (10.0%) were HIV-infected. However, the majority of the samples from the control group lacked HIV status information and average mean age was not calculated in the control group due to missing data.

**Table 1:** Demographic and clinical characteristics of study subjects by HIV status.

**Characteristics**	**HIV-uninfected** ***N* = 70**	**HIV-infected** ***N* = 87**	**Missing data**	**Total (*n*)**
Age^*^ **in years,** **median (IQR)**	61 (50–72)	43 (37–49)	–	–
**ICC**	46 (35.9%)	77 (60.2%)	5 (3.9%)	128
**Non-cancer**	24 (24%)	10 (10%)	66 (66.0%)	100

*IQR, interquartile range; HIV, human immunodeficiency virus; ICC, invasive cervical cancer*.

* *Age data available only for ICC samples*.

### Comparison of Promoter Methylation of Tumor Suppressor Genes in Invasive Cervical Cancer vs. Non-cancer Patients

The status of promoter methylation of four tumor suppressor genes (*RARB, CADM1, DAPK1*, and *PAX1*) in 128 invasive cervical cancer specimens versus 100 specimens of without cancer is shown in Table 2. Interestingly, the patients with invasive cervical cancer showed higher frequency promoter methylation for individual genes: *RARB*, 94.0%; *CADM1*, 76.5%; *PAX1*, 96.5%, and *DAPK1*, 64.1%, compared to the control samples (*RARB*, 50.5%; *CADM1*, 32.6%; *PAX1*, 81.9%, and *DAPK1*, 25.0%), Table 2. Yates-corrected chi-square test results revealed a significant correlation between the methylation rate by gene and invasive cervical cancer presence in all the tumor suppressor genes. Interestingly, *RARB* gene showed the strongest association compared to other tumor suppressor genes (OR = 15.25; 95% CI: 6.06–40.0; *P* < 0.001).

**Table 2:** Promoter methylation frequency (absolute and relative values) by gene and methylation status, in women with and without invasive cervical cancer.

	***RARB***		***CADM***		***DAPKI***		***PAXI***	
**Methylation status**	**ICC (%)**	**Normal cervix (%)**	**ICC (%)**	**Normal cervix (%)**	**ICC (%)**	**Normal cervix (%)**	**ICC (%)**	**Normal cervix (%)**
U	7 (6.0)	47 (49.5)	24 (23.5)	58 (67.4)	42 (35.9)	75 (75.0)	4 (3.5)	17 (18.5)
MU	19 (16.4)	38 (40.0)	16 (15.7)	13 (15.1)	50 (42.7)	23 (23.0)	16 (14.0)	4 (4.3)
M	90 (77.6)	10 (10.5)	62 (60.8)	15 (17.5)	25 (21.4)	2 (2.0)	94 (82.5)	71 (77.2)
M + MU	109 (94.0)	48 (50.5)	78 (76.5)	28 (32.6)	75 (64.1)	25 (25.0)	110 (96.5)	75 (81.5)
Total	116	95	102	86	117	100	114	92
**Comparison: Yates-corrected chi-square value (with** ***P*****), df** **=** **1; OR (95% CI)**								
U vs. MU	5.33 (*0.021*)	3.36 (1.17–9.92)	5.16 (*0.023*)	2.97 (1.14–7.82)	17.84 (*<0.001*)	3.88 (1.199–7.60)	(<*0.001*)^*^	17.00 (3.63–179.69)
U vs. M	85.99 (<*0.001*)	60.43 (19.63–197.4)	39.97 (<*0.001*)	9.99 (4.51–22.48)	(<*0.001*)^*^	22.32 (45.03–98.94)	(*0.001*)^*^	5.63 (1.81–17.45)
MU vs. M	13.64 (<*0.001*)	3.96 (1.81–8.88)	3.36 (*0.016*)	5.72 (1.21–9.39)	(*0.018*)^*^	5.75 (1.25–26.36)	(*0.081*)^*^	0.33 (0.11–1.03)
U vs. [M+MU]	49.50 (<*0.001)*	15.25 (6.06–40.0)	34.82 (<*0.001)*	6.73 (3.38–13.52)	31.62 (<*0.001*)	5.36 (2.86–10.11)	10.88 (<*0.001*)	6.23 (1.87–22.89)

*U, unmethylated promoter; MU, semi-methylated promoter; M, methylated promoter; ICC, invasive cervical cancer; df, degrees of freedom; OR, odds ratio; CI, confidence interval*.

* *Based on Fisher exact test due to insufficient data counts. Italic indicates P values*.

### Cancer, Promoter Methylation, and HIV Status

Binary Logistic Regression analysis was done to determine which factors influence cancer presence. Results showed that cervical cancer was associated with HIV infection (OR = 5.52; 95% CI: 1.23–24.79; *P* = 0.026) and promoter methylation of four tumor suppressor genes, Table 3. *RARB* methylation showed a stronger association with cancer in comparison to the other tested genes (OR = 46.87; 95% CI: 9.61–228.54; *P* < 0.001).

**Table 3:** Logistic regression analysis results.

		**Variables tested in the logistic regression analysis**											
		**ICC vs. non-cancer (*****n*** **=** **228)**		**Cancer stage (*****n*** **=** **128)**		**Promoter methylation**							
						***RARB***		***CADM1***		***DAPKI***		***PAXI***	
		**OR (95% CI)**	***P-*value**	**χ**^**2**^	***P-*value**	**OR (95% CI)**	***P-*value**	**OR (95% CI)**	***P-*value**	**OR (95% CI)**	***P-*value**	**OR (95% CI)**	***P-*value**
**Factors**	***RARB***	46.87 (9.61–228.54)	<0.001	17.32	0.002	–		–		–		–	
	***CADM***	4.64 (1.89–11.35)	0.001	12.68	0.013	–		–		–		–	
	***DAPKI***	4.21 (1.69–10.49)	0.002	5.09	0.279	–		–		–		–	
	***PAXI***	10.04 (2.03–49.67)	0.005	5.21	0.267	–		–		–		–	
	**HIV status**	5.52 (1.23–24.79)	0.026	19.93	0.001	1.53 (0.52–4.47)	0.437	0.73 (0.31–1.74)	0.480	1.15 (0.52–2.55)	0.730	1.95 (0.34–11.20)	0.455
	**HPV-16**	N/A^*^		0.82	0.845	0.00 (0.00–0.00)	0.999	1.79 (0.33–9.77)	0.500	1.15 (0.26–5.10)	0.854	10.48 (0.76–144.01)	0.079
	**HPV-18**	N/A^*^		0.95	0.814	2.58 (0.29–22.94)	0.395	1.62 (0.42–6.33)	0.485	0.44 (0.15–1.33)	0.147	2.79 (0.18–42.96)	0.463
	***Other*** **hr-HPV**	N/A^*^		4.91	0.179	1.13 (0.20–6.41)	0.888	2.40 (0.52–11.09)	0.262	2.50 (0.79–7.86)	0.118	1.51 (0.13–18.09)	0.746
	**Age**	N/A^*^		1.49	0.684	1.12 (1.01–1.26)	0.037	1.02 (0.96–1.08)	0.532	1.05 (1.00–1.10)	0.040	1.02 (0.93–1.12)	0.625

*HIV, human immunodeficiency virus; HPV, human papillomavirus; hr, high risk; ICC, invasive cervical cancer; OR, odds ratio; CI, confidence interval; χ^2^, chi square value; N/A, not applicable*.

* *Genotypes available only for ICC specimens*.

*Sample sizes differ slightly between analyses because of missing values and/or efficiency of molecular test*.

### Gene (*RARB* and *DAPK1*) Promoter Methylation Is Associated With Age in Patients With Invasive Cervical Cancer

We further evaluated if the promoter methylation was associated with age in the invasive cervical cancer group. After removal of non significant variables (HIV status and high-risk HPV genotypes) results showed that there was a positive association of *RARB* and *DAPK1* promoter methylation with age (OR = 1.12; 95% CI: 1.01–1.26; *P* = 0.037 and OR = 1.05; 95% CI: 1.00–1.10; *P* = 0.040, respectively), Table 3, whereas no association was found for the other two genes. Note that there was only a weak non-significant association (*P* = 0.079) of *PAX1* methylation with HPV-16 genotype presence alone or in combination with *other* high-risk HPV genotypes.

### Assessment of Promoter Methylation Rate by HIV Infection and Cervical Cancer Stage

Methylation rate by HIV status did not vary significantly in all the four tumor suppressor genes, Table 3. Instead, the analysis showed that cancer stage was affected by HIV status (χ^2^ = 19.93; *P* = 0.001), *RARB* (χ^2^ = 7.32; *P* = 0.002), and *CADM1* (χ^2^ = 12.68; *P* = 0.013) methylation status.

### Cancer Status According to the Number of Methylated Promoter Genes

**Figure 1:**
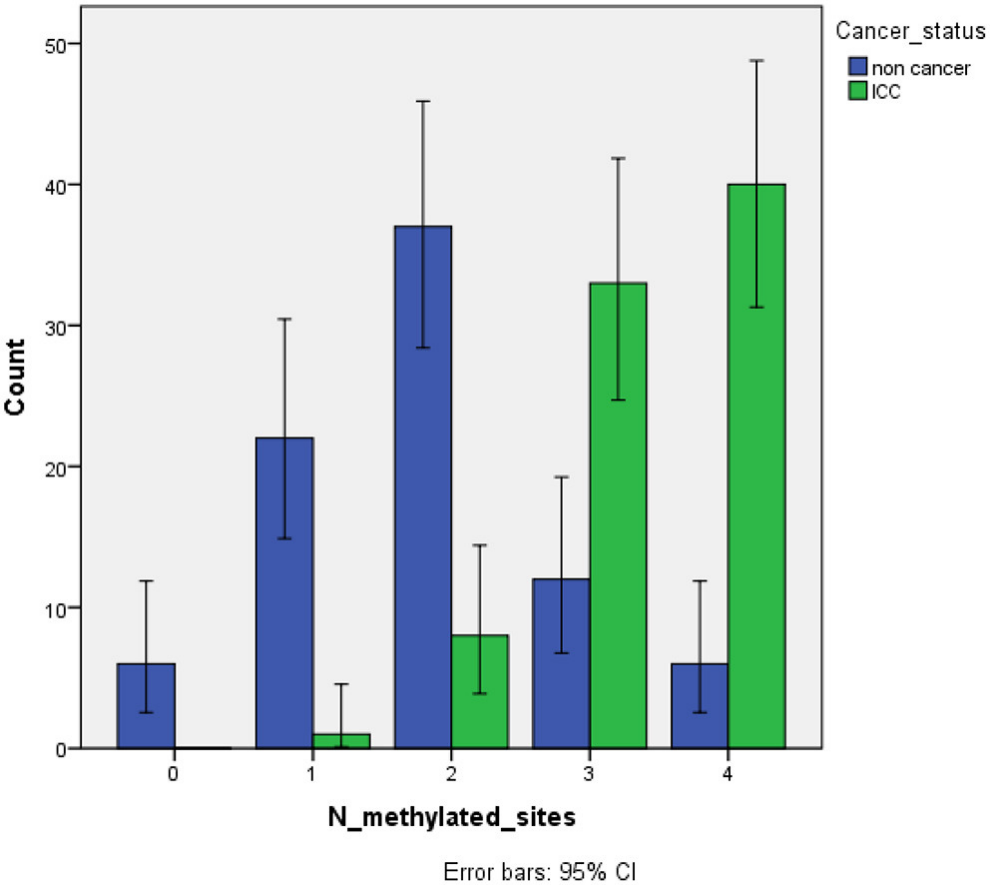
Cervical cancer status according to the number of methylated promoters. ICC, invasive cervical cancer. In *x-*axis a score of 0 indicates “no methylation,” a score of 1 indicates “1 methylated site,” a score of 2 indicates “2 methylated sites,” a score of 3 indicates “3 methylated sites,” and a score of 4 “4 methylated sites”. Non cancer (*n* = 83); invasive cervical cancer (*n* = 82).

Finally, the possible association among a number of promoter methylated sites (from 0 to 4) and cancer status was tested in 82 cervical cancer cases versus 83 non-cancer control (Figure 1). The analysis showed a higher risk of cervical cancer according to the increased number of methylated promoter genes (OR = 6.20; 95% CI: 3.66–10.51; *P* < 0.001).

## Discussion

In a high HIV endemic setting such as Botswana, cervical cancer is the leading cause of morbidity and mortality among women. Our study explored promoter methylation of four tumor suppressor genes in the cervical epithelium of HPV positive women with and without invasive cervical cancer in relation to their HIV status. Previous studies have focused on promoter methylation of different genes in cervical cancer but there are few done in relation to HIV. We found a significant association between cancer, the presence of methylated promoters and the number of methylated sites. Nevertheless, promoter methylation among cancer subjects was independent of patient's HIV status in our study. We selected four tumor suppressor genes, consistently demonstrated to be affected in cervical cancer by reviewing the existing literature. Specifically, we characterized aberrantly methylated host promoter genes *(RARB, CADM1, DAPK1*, and *PAX*1) (28, 30, 34, 35) and their possible association with: invasive cervical cancer, cervical cancer stage, HIV status, age, high-risk HPV genotypes. We observed a significant higher frequency of subjects having promoter methylation signals at the four genes, *RARB (*94.0%), *CADM1* (76.5%), *DAPK1 (*64.1%), and *PAX1* (96.5%), in patients with invasive cervical cancer compared to the subjects without cancer (Table 2). Our findings are consistent with other studies. For example, a study by Virmani et al. (44) reported a high frequency of promoter methylation among the US population. Concordant results were also observed in a study by Mao et al. (45) and Steenbergen et al. (46), where hypermethylation of the *CADM1* gene promoter was reported. Additionally, the promoter methylation rate of the same gene was 83% in cervical carcinoma cases in another study (41). Interestingly, the promoter methylation of *DAPK1* is known to be associated with aggressive and metastatic phenotype in many tumor types (47). The promoter methylation rate of *DAPK1* in the present study was higher compared to what was found by Narayan et al. (30) (43.3%) and Dong et al. (48) (51%). A reason for the slight differences in our respective results may be due to different methods used to analyze methylation pattern in the two studies and the use of different primers detecting different CpGs within the same CpG island. Moreover, differences in ethnicity should also be taken into account (30, 48).

Among the four genes, *RARB* gene promoter methylation showed the strongest association to cancer when compared to other genes at the Binary Logistic Regression analysis (OR = 15.25; 95% CI: 6.06–40; *P* < 0.001; Table 2). *RARB* promoter methylation has been shown to be an early event in multistage cervical carcinogenesis with overall high frequencies of promoter methylation reported in cervical cancer specimens (32, 44). Our data further confirm the possible role of promoter methylation of *RARB, CADM1, DAPK1*, and *PAX1* in cervical cancer tumorigenesis. Our results corroborate a review which summarized the results of 51 published studies on methylation analyses performed in cervical tissues and cells, which suggested that the combination of *RARB, CADM1*, and *DAPK1* genes is the most promising methylation panel for obtaining an appropriate predictive tool of cervical cancer screening (28). Again, a study by Narayan et al. (30) has found that *RARB* promoter methylation was associated with cervical cancer prognosis, i.e., 80% of the patients with *RARB* hypermethylation either died of cancer or only partly responded to treatment. Interestingly, Choi et al. (49), have observed the inverse relationship between the levels of RARB protein expression and expression of squamous cervical cancer antigen which is an early tumor marker for diagnosing cervical cancer and monitoring responses to treatment in the event of relapse. We are limited by the lack of data on squamous cervical cancer antigen to know the association with RARB protein expression.

We observed that, among the cancer subjects, methylation rate according to HIV status did not vary significantly in all four tumor suppressor genes. This may be consistent with the evidence that cervical precancer in HIV-positive women is associated with high levels of methylation of high-risk HPV genome, thus raising the possibility that HIV influences the methylation of HPV viral genome rather than the host genome in the rapid progression of cancer (50). These findings might even help to understand the clinical behavior and treatment response of cervical cancer patients with HIV infection as shown by Ferreira et al. (51). Despite the proof that previous work described a relation between HIV and methylation of host cell genes, resulting in an upregulation of DNA methyltransferases expression and activity in HIV infected cells (52, 53), medical community continues to explore the effect of HIV on cervical premalignant lesions with subsequent progression to cancer. However, the lack of correlation between HIV and methylation in our study may be due to: (i) the control samples lacked HIV status information; (ii) we were unable to control for age (known to affect methylation rate in several genes) among the control samples; (iii) HIV is generally well managed in Botswana and this was also true for this study cohort (13), possibly implying a minor impact on methylation homeostasis also based on the length of ART; (iv) the methylation status was assessed for only four promoter regions, then neglecting other possible targets among several interconnected and regulatory genes. Another interesting result is the association among number of promoter methylated sites and cancer status where we found a higher rate of invasive cervical cancer according to the increased number of hypermethylated promoter regions.

We also found that invasive cervical cancer and promoter methylation of *RARB* and *DAPK1* gene was associated with age, while no association was found for *CADM1* and *PAX1* gene. Conversely, the study by Narayan et al. (25) demonstrated that age had no influence on overall frequency of promoter methylation for *RARB* and *DAPK1*. In particular, they did show that *RARB* gene promoter was more frequently methylated in younger patients (34.7% in below 50 years compared to 21.2% in above 50 years) (25). This contrasting result may be attributed to different methods employed, including region of the gene used for analysis, and ethnic differences (25, 43). In this study we also found that promoter methylation status for all genes taken individually was not associated with high-risk HPV genotype presence (HPV-16 and/or HPV-18 and/or *other* high risk-HPV) on patients with invasive cervical cancer. Only a weak non-significant association of *PAX1* promoter methylation with HPV-16 genotype was found. Finally, it should be highlighted that this unique cohort of cervical cancer patients, in the high HIV setting of Botswana, provided an opportunity to explore the interplay between HIV, HPV, and cervical cancer, in a context of a human genetic background that shows peculiar attributes. In fact, Botswana is home to a population of which genetic structure has potential implications on susceptibility and resistance to infectious diseases but also treatment outcomes (54–57), and has never been analyzed for epigenetic studies in cancer progression.

Although the study had several merits, there were limitations on a few fronts that warrant discussion. The controls were not age matched to cases due to lack of data, therefore we could not control for potential confounders. Some of the comparisons between cancer and non-cancer subjects could also not be performed due to insufficient HIV status from control patients. The expression of all genes validated for methylation in this study was not measured. HIV viral load and HPV copy number were also not quantified. Another limitation of our study was the limited number of genes (four) analyzed. While, providing data on wider spectrum methylation, could have shed more information on the role of epigenetic process on cervical cancer progression.

## Conclusion

The current study presents novel initial data showing that promoter methylation in HIV infected women with cervical cancer is not significantly different from the HIV uninfected women with cervical cancer. Genome wide methylation profile studies are needed to completely shed light on the role of HIV in methylation of host genes among cancer subjects leading to cervical cancer progression. In addition, this study further substantiated the previous studies results of overall high frequency of methylation rate in promoter regions of *RARB, CADM1, DAPK1*, and *PAX*1 genes in cervical cancer subjects. Finally, the number of methylated sites in four genes showed a higher risk of cervical cancer.

## Data Availability Statement

The original contributions presented in the study are included in the article/Supplementary Material, further inquiries can be directed to the corresponding author/s.

## Ethics Statement

The research was approved by Institutional Review Board (IRB) at the University of Botswana, the Human Resource Development Council at the Botswana Ministry of Health and Wellness and the University of Pennsylvania's IRB. Written informed consent for participation was not required for this study in accordance with the national legislation and the institutional requirements.

## Author Contributions

LT: carried out the experiments, performed statistical analysis, and wrote the manuscript with support from all authors. SGr: conceived the original idea and supervised the work. NZ: conceived the original idea. ESR: involved in planning and supervised the work. SGa: involved in planning and supervised the work. SM: involved in planning, supervised the work, provided overall direction, and planning of the performed the analysis. IK: helped supervise the project, provided overall direction, and planning of the work. GMP: conceived the study and was in charge of overall direction and planning, performed statistical analysis, and wrote the manuscript. MN: conceived the original idea, helped supervise the project, helped map up the overall direction, planning of the work, and performed the analysis. All authors provided critical feedback and helped shape the research, analysis, and manuscript.

## Conflict of Interest

The authors declare that the research was conducted in the absence of any commercial or financial relationships that could be construed as a potential conflict of interest.

## Supplementary Material

Supplementary Figure 1 Graphical representation of the regions used for bisulfite based methylation measurement in all four genes.

Supplementary Table 1 Gene name, primer sequences, and annealing temperatures for the MS-PCR analyses. M, methylated; U, unmethylated; Ta, annealing temperature; bp, base pair.

Supplementary Table 2 **(A)** Designed methylated touch-down MS-PCR for detection of promoter methylation of four genes. ^*^Ta is reduced 0.5°C each cycle. **(B)** Designed unmethylated touch-down MS-PCR for detection of promoter methylation of four genes. ^*^Ta is reduced 0.5°C each cycle.
