# Supplementary material for: Promoter Hypermethylation Analysis of Host Genes in Cervical Cancer Patients With and Without Human Immunodeficiency Virus in Botswana
Source: Front Oncol. 2021 Feb 26;11:560296. doi: 10.3389/fonc.2021.560296 (PMC7952881; doi:10.3389/fonc.2021.560296)
Supplement: Supplementary Figure 1 — Graphical representation of the regions used for bisulfite based methylation measurement in all four genes. [file Table_3.docx]

Supplementary material

1. **B)**


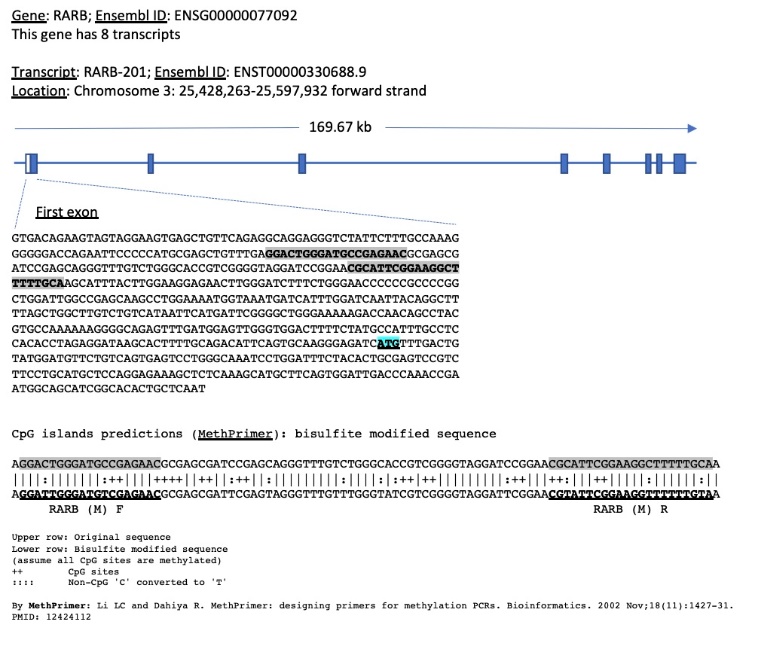

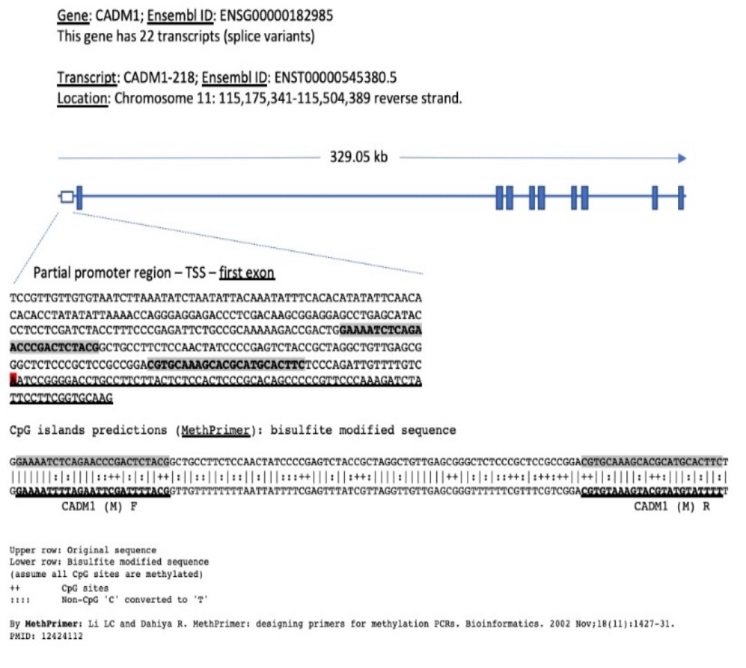


**C) D)**


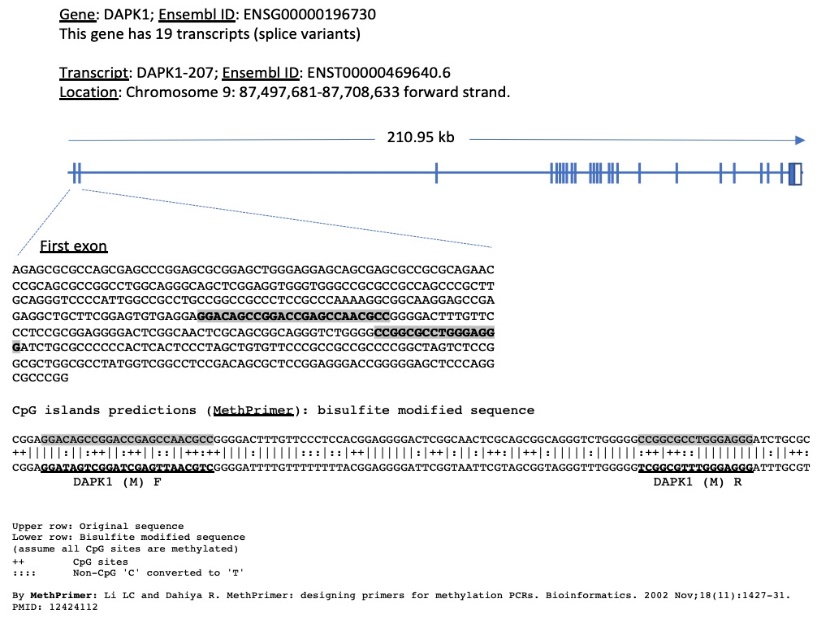

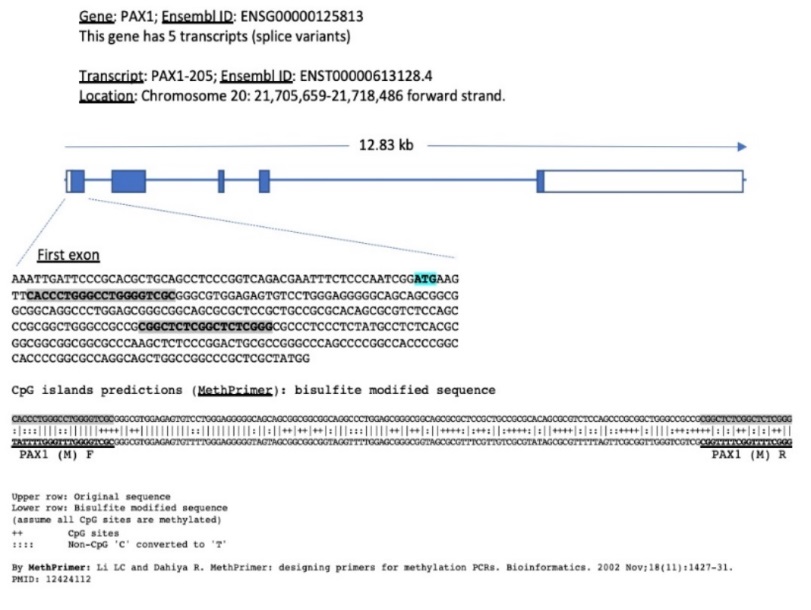


**Supplementary Figure 1.** Graphical representation of the regions used for bisulfite based methylation measurement in all four genes.
