# Supplementary material for: Promoter Hypermethylation Analysis of Host Genes in Cervical Cancer Patients With and Without Human Immunodeficiency Virus in Botswana
Source: Front Oncol. 2021 Feb 26;11:560296. doi: 10.3389/fonc.2021.560296 (PMC7952881; doi:10.3389/fonc.2021.560296)
Supplement: Supplementary Table 1 — Gene name, primer sequences, and annealing temperatures for the MS-PCR analyses. M, methylated; U, unmethylated; Ta, annealing temperature; bp, base pair. [file Table_1.docx]

| **Gene name** | **Primer sequence** | **Ta °C** | **Size (bp)** | **Reference** |
| --- | --- | --- | --- | --- |
| ***RARB*** | **(M)**  F (GGATTGGGATGTCGAGAAC); R (TACAAAAAACCTTCCGAATACG)  **(U)**  F (AGGATTGGGATGTTGAGAATG); R (TTACAAAAAACCTTCCAAATACA) | 64  54 | 93  95 | Chan et al. [40] |
| ***CADM1*** | **(M)**  F (GAAAATTTTAGAATTCGATTTTACG); R (AAAATACATACGTACTTTACACG)  **(U)**  F (GAAAATTTTAGAATTTGATTTTATG); R (AAAAAAATACATACATACTTTACACA) | 64  54 | 114  114 | Overmeer et al. [41] |
| ***DAPK1*** | **(M)**  F (GGATAGTCGGATCGAGTTAACGTC)**;** R (CCCTCCCAAACGCCGA)  **(U)**  F (GGAGGATAGTTGGATTGAGTTAATGTT); R (CAAATCCCTCCCAAACACCAA) | 60  60 | 98  106 | Overmeer et al. [41] |
| ***PAX1*** | **(M)**  F (TATTTTGGGTTTGGGGTCGC); R (CCCGAAAACCGAAAACCG)  **(U)**  F (GTTTATTTTGGGTTTGGGGTTGTG); R  (CACCCAAAAACCAAAAACCAC) | 64  53 | 153  158 | Su et al. [58] |
|  |  |  |  |  |

**Supplementary Table 1**. Gene name, primer sequences and annealing temperatures for the MS-PCR analyses.

Abbreviations: M= methylated, U= unmethylated; Ta= annealing temperature, bp=base pair

REFERENCES

58. Su HY, Lai HC, Lin YW, Chou YC, Liu CY, Yu MH. An epigenetic marker panel for screening and prognostic prediction of ovarian cancer*. Int J Cancer*. (2008) 124:387–93. doi: 10.1002/ijc.23957
