# Supplementary material for: Promoter Hypermethylation Analysis of Host Genes in Cervical Cancer Patients With and Without Human Immunodeficiency Virus in Botswana
Source: Front Oncol. 2021 Feb 26;11:560296. doi: 10.3389/fonc.2021.560296 (PMC7952881; doi:10.3389/fonc.2021.560296)
Supplement: Supplementary Table 2 — (A) Designed methylated touch-down MS-PCR for detection of promoter methylation of four genes. *Ta is reduced 0.5°C each cycle. (B) Designed unmethylated touch-down MS-PCR for detection of promoter methylation of four genes. *Ta is reduced 0.5°C each cycle. [file Table_2.docx]

|  | ***RARB*** | | | | ***CADM1*** | | | ***DAPK1*** | | | ***PAX1*** | | |
| --- | --- | --- | --- | --- | --- | --- | --- | --- | --- | --- | --- | --- | --- |
| **Step** | **Stage** | **Temp °C** | **Time** | **Cycles** | **Temp °C** | **Time** | **Cycles** | **Temp °C** | **Time** | **Cycles** | **Temp °C** | **Time** | **Cycles** |
| **1** | Initial Denature | 95 | 5min |  | 95 | 5min |  | 95 | 5min |  | 95 | 5min |  |
| **2** | Denature | 95 | 30sec |  | 95 | 30sec |  | 95 | 30sec |  | 95 | 30sec |  |
| **3** | Anneal* | 69-64 | 1min |  | 64-58 | 1min |  | 65-60 | 1min |  | 69-64 | 1min |  |
| **4** | Extension | 72 | 1min |  | 72 | 1min |  | 72 | 1min |  | 72 | 1min |  |
| **5** | Repeat step 2-4 |  |  | 10 |  |  | 10 |  |  | 10 |  |  | 10 |
| **6** | Denature | 95 | 30sec |  | 95 | 30sec |  | 95 | 30sec |  | 95 | 30sec |  |
| **7** | Anneal | 64 | 30sec |  | 64 | 30sec |  | 60 | 30sec |  | 64 | 30sec |  |
| **8** | Extension | 72 | 1min |  | 72 | 1min |  | 72 | 1min |  | 72 | 1min |  |
| **9** | Repeat  Step 6 |  |  | 30 |  |  | 30 |  |  | 30 |  |  | 30 |
| **10** | Extension | 72 | 10min |  | 72 | 10min |  | 72 | 10min |  | 72 | 10min |  |

**Supplementary Table 2A:** Designed methylated touch-down MS-PCR for detection of promoter methylation of four genes.

*Ta is reduced 0.5**°**C each cycle.

|  | ***RARB*** | | | | ***CADM1*** | | | ***DAPK1*** | | | ***PAX1*** | | |
| --- | --- | --- | --- | --- | --- | --- | --- | --- | --- | --- | --- | --- | --- |
| **Step** | **Stage** | **Temp** | **Time** | **Cycles** | **Temp** | **Time** | **Cycles** | **Temp** | **Time** | **Cycles** | **Temp** | **Time** | **Cycles** |
| **1** | Initial Denature | 95 | 5min |  | 95 | 5min |  | 95 | 5min |  | 95 | 5min |  |
| **2** | Denature | 95 | 30sec |  | 95 | 30sec |  | 95 | 30sec |  | 95 | 30sec |  |
| **3** | Anneal* | 59-54 | 1min |  | 59-54 | 1min |  | 65-60 | 1min |  | 58-53 | 1min |  |
| **4** | Extension | 72 | 1min |  | 72 | 1min |  | 72 | 1min |  | 72 | 1min |  |
| **5** | Repeat step 2-4 |  |  | 10 |  |  | 10 |  |  | 10 |  |  | 10 |
| **6** | Denature | 95 | 30sec |  | 95 | 30sec |  | 95 | 30sec |  | 95 | 30sec |  |
| **7** | Anneal | 54 | 30sec |  | 54 | 30sec |  | 60 | 30sec |  | 53 | 30sec |  |
| **8** | Extension | 72 | 1min |  | 72 | 1min |  | 72 | 1min |  | 72 | 1min |  |
| **9** | Repeat  Step 6 |  |  | 30 |  |  | 30 |  |  | 30 |  |  | 30 |
| **10** | Extension | 72 | 10min |  | 72 | 10min |  | 72 | 10min |  | 72 | 10min |  |

**Supplementary Table 2B:** Designed unmethylated touch-down MS-PCR for detection of promoter methylation of four genes.

*Ta is reduced 0.5**°**C each cycle.
